# Genetic Mutations in *TNFSF11* Were Associated With the Chronicity of Hepatitis C Among Chinese Han Population

**DOI:** 10.3389/fmed.2021.743406

**Published:** 2021-10-01

**Authors:** Peng Huang, Yu-Qing Hou, Jing-Jing Wu, Yi-Di Wang, Xiang-Yu Ye, Feng Zang, Rong-Bin Yu, Sheng Yang

**Affiliations:** ^1^Department of Epidemiology, Center for Global Health, School of Public Health, Nanjing Medical University, Nanjing, China; ^2^Department of Environmental Health, Yangzhou Center for Disease Control and Prevention, Yangzhou, China; ^3^Infection Management Office of the First Affiliated Hospital of Nanjing Medical University, Nanjing, China; ^4^Department of Biostatistics, School of Public Health, Nanjing Medical University, Nanjing, China

**Keywords:** gene polymorphism, hepatitis C virus, TNFSF11, chronicity, bioinformatics

## Abstract

**Background:** Recently, several studies have reported that the host immune response can be related to the RANKL/RANK/OPG signaling pathway. However, the associations of *TNFSF11, TNFRSF11A*, and *TNFRSF11B* gene polymorphisms in the RANKL/RANK/OPG pathway with hepatitis C virus (HCV) infection outcomes remain unclear.

**Methods:** In this case-control study, 768 persistent HCV infection and 503 spontaneous HCV clearance cases, and 1,259 control subjects were included. The Taman-MGB probe method was utilized to detect *TNFSF11* rs9525641, *TNFRSF11A* rs8686340, and *TNFRSF11B* rs2073618 genotypes. The distribution of three single nucleotide polymorphisms (SNPs) genotypes was analyzed using stata14.0.

**Results:** SNPs rs9525641, rs8086340, and rs2073618 genotype frequencies followed the Hardy-Weinberg natural population equilibrium (*p* = 0.637, 0.250, and 0.113, respectively). Also, rs9525641 was significantly associated with HCV chronicity risk in recessive (OR = 1.203, 95% CI: 1.018–1.420, *p* = 0.030) and additive models (OR = 1.545, 95% CI: 1.150–2.075, *p* = 0.004). The stratified analysis showed that rs9525641 variant genotypes were associated with HCV chronicity among people older than 50 years (OR =1.562, 95% CI: 1.079–2.262, *p* = 0.018), females (OR = 1.667, 95% CI: 1.145–2.429, *p* = 0.008), ALT <40 U/L (OR = 1.532, 95% CI: 1.074–2.286, *p* = 0.018), and AST < 40 U/L (OR = 1.552, 95% CI: 1.095–2.201, *p* = 0.014).

**Conclusion:** *TNFRSF11* rs9525641 was significantly associated with HCV chronicity in the Chinese population.

## Introduction

With the introduction of all-oral direct-actingantiviral therapy, a substantial breakthrough has been made in chronic HCV infection treatment during the past decades (1). However, due to HCV's significant heterogeneity and high variability, virus reinfection following successful treatment remains an important public health problem (2). The HCV pathogenesis and progression are complex and interact with its biological characteristics, environmental behavior factors, host immunity, and genetic background.

The activation of NF-κB and NF-κB-dependent inflammatory pathways are important to chronic HCV infection and its related cirrhosis and HCC. The NF-κB ligand (RANKL) receptor activator, a 316-amino acid transmembrane protein, is highly expressed in different immune cells including T or dendritic cells. RANKL can be induced by inflammatory factors such as interleukin 1, tumor necrosis factor α, and transforming growth factor β (3). Besides, RANK and osteoprotegerin (OPG) are RANKL receptors, and the RANKL/RANK/OPG pathway is important for cellular immune responses such as cell death and proliferation, inflammation, and immunity (4). The communication pathways mediated by TNFSF/TNFRSF are essential for numerous developmental, homeostatic, and stimulus-responsive processes. Both innate and adaptive immune cells are controlled by TNFSF/TNFRSF members in a manner that is crucial for the coordination of various mechanisms driving either co-stimulation or co-inhibition of the immune response (5). Different cellular immune responses can be triggered by genetic differences between different individuals. During the past decades, many studies have identified that immune cytokines SNPs were significantly associated with HCV spontaneous clearance and virological response, except for viral and environmental factors (6–9). Studies have shown that multiple TNFSF and TNFRSF gene SNPs are related to autoimmune diseases, suggesting that these SNPs play an important role in immunity. rs8086340 and rs2073618 are closely related to the occurrence of rheumatoid arthritis (10), and rs9525641 may affect the susceptibility and severity of AS disease (11). However, no research has addressed *TNFSF* and *TNFRSF* genetic polymorphisms' impacts on HCV-related chronic liver diseases. Considering that China has the largest number of HCV infections (about 10 million patients), we examined the relationships between OPG-RANKL-RANL pathway genes SNPs rs9525641, rs2073618, and rs8686340, and HCV infection outcomes in a high-risk Chinese population.

## Methods

### Study Participants

This study included three HCV infection high-risk groups. In the present study, 2,800 subjects were recruited from 2008 to 2016, including 722 hemodialysis patients from 9 hemodialysis centers in southern China, 459 drug users from a Nanjing compulsory detoxification center, and 1,619 paid blood donors from 6 Zhenjiang villages. All research objects voluntarily signed the informed consent. The exclusion criteria were: (1) patients under 18 years and over 80 years; (2) patients with interferon treatment history; (3) patients co-infected with HBV and HIV; (4) patients who suffer from autoimmune diseases or malignant tumors; (5) patients with other liver diseases. All patients were diagnosed with patient's clinical symptoms and biochemical examination indicators by experienced doctors, strictly following international standards. Patients were grouped according to their HCV antibodies and viral load. The participants were categorized into three groups: (1) A, uninfected control (anti-HCV and HCV RNA negative); (2) B, spontaneous HCV clearance (anti-HCV positive and HCV RNA negative); (3) C, persistent HCV infection cases (anti-HCV and HCV RNA positive).

This study was conducted strictly under the “Declaration of Helsinki” and was approved by the Ethics Committee of Nanjing Medical University (2017445).

We interviewed each participant with trained personnel and collected demographic data and environmental exposure history information through a structured survey. All participants were informed and agreed to participate in this study before recruitment.

### Data and Blood Sample Collection

After the interview, we collected venous blood samples (~10 mL) from each participant, separated the plasma and white blood cells, and stored them at −80°C until assays. The detection of subjects' anti-HCV antibodies, HCV RNA, and HCV genotypes was performed with Jurong City People's Hospital and Yixing City People's Hospital, using third-generation enzyme-linked immunosorbent assay (ELISA) (Architect Anti-HCV assay, Abbott Laboratories, Abbott Park, IL, USA), Trizol LS reagent (Takara Biotech, Tokyo, Japan) and the murex HCV serotype ELISA kit (Abbott, Wiesbaden, Germany), respectively. The HCV RNA load detection limit was 1 × 10^3^ IU/ml and all serological tests were performed with the same analytical systems.

### SNP Selection and Genotyping

Candidate *TNFSF11*/*TNFRSF11A*/*TNFRSF11B* gene Tag SNPs were selected by searching the 1,000 Genomes Project (http://www.1000genomes.org/) or the HapMap (http://www.hapmap.org/) databases. The selected SNPs were filtered with the following criteria: (1) minor allele frequency >5% in Han Chinese population, acquired from the Haploview software (version 4.2; Broad Institute, Cambridge, MA, USA); (2) Hardy-Weinberg equilibrium test *p* ≥ 0.05; (3) reported SNPs from previous studies associated with immune-related disorders; (4) combined bioinformatics data from Regulome DB (http://regulome.stanford.edu/). Finally, three SNP candidates (rs9525641, rs8086340, and rs2073618) were chosen for genotyping. Primers and probes are presented in Supplementary Table 1.

Genomic DNA was isolated from subjects' peripheral blood leukocytes using protease K digestion, phenol-chloroform extraction, and ethanol precipitation. SNPs genotyping was performed with a Taman allelic discrimination assay on the LightCycler® 480 IIReal-Time PCR System (Roche, Switzerland). All genotyping was performed without knowing the subjects' case or control status. Each SNP accordance rate was 100% for the repeated experiments of 10% random samples. Additionally, the genotyping success rates for these polymorphisms were above 95%. All tests were carried out following the manufacturer's instructions and were performed with the same analytical systems.

### *In silico* Analysis

The function of SNPs was predicted using the Regulome DB online database, HaploReg database (https://pubs.broadinstitute.org/mammals/haploreg/haploreg.php) and Vienna RNA Web Servers (http://rna.tbi.univie.ac.at/cgi-bin/RNAWeb/Suite/RNAfold.cgi). The Regulome DB online database annotated SNPs with known and predicted regulatory elements in *Homo sapiens* genome intergenic regions was used to obtain SNPs' Regulome DB scores. Different Regulome DB score represents different combinations of the above and detailed information on all Regulome DB scores (Supplementary Table 2). The HaploReg database can be used for exploring chromatin states, conservation, and regulatory motif alterations within a set of genetically linked variants. Moreover, RNA secondary structures were predicted using the Vienna RNA Web Servers based on its latest package (Version 2.3.1).

### Statistic Analysis

The demographic and clinical data distribution among the three groups was compared using the χ^2^ test. HWE was assessed among control subjects by the goodness-of-fit χ^2^ test. Logistic regression, with age, gender, and high-risk population adjustments, was used to analyze the relationship between the three SNPs and HCV infection outcome according to four genetic models, showing odds ratio and 95% confidence intervals. Hierarchical analysis was used to control confounding factors' effects on the results and Q tests were used to determine heterogeneity between subgroups. Statistical analyzes were performed using stata14.0, and a two-sided *p* < *0.05* was considered statistically significant.

## Results

### Demographic and Clinical Characteristics of Participants

According to the HCV antibody and RNA, subjects were divided into three groups. The demographic and clinical characteristics distribution among the HCV-uninfected control, the spontaneous HCV clearance, and the persistent infection groups are presented in Table 1. No significant differences in age or gender distribution among groups were detected (*p* = 0.185 and 0.095, respectively). On the other hand, alanine aminotransferase (ALT), aspartate aminotransferase (AST), infection routes, and HCV genotype differed (*p* < 0.001).

**Table 1:** Demographic and clinical characteristics among HCV control, spontaneous clearance, and persistent infection groups.

**Variables**	**Group A (%)**	**Group B (%)**	**Group C (%)**	***P***
	***n* = 1,529**	***n* = 503**	***n* = 768**	
Age (years)				0.185
<50	561 (36.69)	193 (38.37)	312 (40.63)	
≥50	968 (63.31)	310 (61.63)	456 (59.38)	
Gender				0.095
Male	615 (40.22)	194 (38.57)	273 (35.55)	
Female	914 (59.78)	309 (61.43)	495 (64.45)	
ALT (U/L)				<0.001
<40	1,437 (94.91)	393 (78.29)	443 (57.83)	
≥40	77 (5.09)	109 (21.71)	323 (42.17)	
AST (U/L)				
<40	1,438 (95.11)	401 (81.17)	454 (59.97)	<0.001
≥40	74 (4.89)	93 (18.83)	303 (40.03)	
High-risk population				<0.001
HD	555 (36.30)	91 (18.09)	76 (9.90)	
IVDU	181 (11.84)	138 (27.44)	140 (18.23)	
PBD	793 (51.86)	274 (54.47)	552 (71.88)	
HCV genotype				<0.001
1b	–	42 (26.25)	223 (46.07)	
Non-1b	–	118 (73.75)	261 (53.93)	

*Group A: controls; Group B: spontaneous clearance subjects; Group C: persistent infection patients.*

*Non-1b means viral strains other than 1b, including genotype 1a, 2, and 3 (either solely or mixed infection).*

*HCV, hepatitis C virus; SD, standard deviation; ALT, alanine transaminase; AST, aspartate transaminase; HD, hemodialysis patients; IVDU, Intravenous drug user; PBD, paid blood donors*.

SNPs rs9525641, rs8086340, and rs2073618 genotype frequencies in the HCV-uninfected control followed the Hardy-Weinberg equilibrium (*p* = 0.637, 0.250, and 0.113, respectively). This indicated that the control was a representative group.

### Associations Between SNP Candidates and HCV Infection Outcomes

The genotype distribution rs9525641, rs8086340, and rs2073618 among groups are shown in Table 2. To analyze the association between these SNPs and HCV infection susceptibility, we first combined patients from groups B and C in an HCV-infected group and compared it with the control (A). However, no significant association was observed in the logistic regression analyses between these three SNPs and HCV infection susceptibility (*p* > 0.05). To determine the association between these SNPs and HCV chronicity, the B group was selected as a control and compared to C. The regression analysis of a co-dominant model—corrected for age, gender, and high-risk population—showed that patients carrying the rs9525641-C gene were significantly associated with HCV chronic diseases (adjusted OR = 1.518, 95% CI: 1.079–2.136, *p* = 0.017), as for a recessive (adjusted OR = 1.203, 95% CI: 1.018–1.420, *p* = 0.030), and additive models (adjusted OR = 1.545, 95% CI: 1.150–2.075, *p* = 0.004). However, no correlation was observed between the two other genotypes and HCV infection chronicity (*p* > 0.05).

**Table 2:** Genotypes distributions of three SNPs among persistent infection, spontaneous clearance and control group.

**SNPs (genotype)**	**Group A *n* (%)**	**Group B *n* (%)**	**Group C *n* (%)**	**OR(95%CI)^**a**^**	***P* ^**a**^**	**OR (95%CI)^**b**^**	***P* ^**b**^**
	***n* = 1,529**	***n* = 503**	***n* = 768**				
*rs9525641*				0.037			
TT	406 (26.71)	144 (29.63)	209 (27.68)	1.00	–	1.00	–
TC	768 (50.53)	260 (53.50)	365 (48.34)	0.946 (0.788–1.137)	0.556	0.973 (0.743–1.274)	0.842
CC	346 (22.76)	82 (16.87)	181 (23.97)	0.892 (0.715–1.114)	0.313	**1.518 (1.079**–**2.136)**	**0.017**
Dominant model				0.930 (0.782–1.105)	0.409	1.104 (0.855–1.425)	0.448
Recessive model				0.925 (0.767–1.115)	0.412	**1.545 (1.150**–**2.075)**	**0.004**
Additive model				0.945 (0.846–1.055)	0.313	**1.203 (1.018**–**1.420)**	**0.030**
*rs8086340*				0.267			
GG	621 (41.40)	212 (43.71)	332 (43.92)	1.00	–	1.00	–
GC	704(46.93)	209 (43.09)	322 (42.59)	0.878 (0.743–1.036)	0.124	0.987 (0.769–1.265)	0.916
CC	175 (11.67)	64 (13.20)	102 (13.20)	1.140 (0.887–1.465)	0.306	1.024 (0.713–1.472)	0.896
Dominant model				0.929 (0.794–1.086)	0.355	0.995 (0.788–1.257)	0.970
Recessive model				1.219 (0.963–1.543)	0.100	1.031 (0.733–1.450)	0.860
Additive model				1.007 (0.898–1.130)	0.902	1.005 (0.850–1.188)	0.952
*rs2073618*				0.149			
GG	854 (56.33)	266 (53.41)	409 (53.46)	1.00	–	1.00	–
GC	552 (36.41)	207 (41.57)	305 (39.87)	1.131 (0.962–1.330)	0.137	0.933 (0.735–1.185)	0.572
CC	110 (110)	25 (5.02)	51 (6.67)	0.841 (0.613–1.155)	0.286	1.318 (0.791–2.197)	0.289
Dominant model				1.082 (0.927–1.264)	0.316	0.975 (0.774–1.227)	0.827
Recessive model				0.799 (0.586–1.090)	0.157	1.358 (0.823–2.239)	0.230
Additive model				1.015 (0.896–1.149)	0.817	1.029 (0.851–1.243)	0.770

*CI, confidence interval; HCV, hepatitis C virus; OR, odds ratio; SNP, single nucleotide polymorphism.*

*Group A: controls; Group B: spontaneous clearance subjects; Group C: persistent infection patients. Group (B+C): Infected individuals.*

*^a^The P-value, OR and 95% CIs of Group (B + C) vs. Group A were calculated on the basis of the logistic regression model, adjusted by gender, age, and high-risk population.*

*^b^The P-value, OR and 95% CIs of Group C vs. Group B were calculated on the basis of the logistic regression model, adjusted by gender, age, high-risk population.*

*Bold type indicates statistically significant results*.

### Stratified Analysis

To control age, gender, high-risk population, and HCV genotypes bias in each population, we performed a stratified analysis to explore the association between the rs9525641 genotype and HCV chronicity using a recessive model (Table 3). Results showed that the rs9525641 variant genotypes were significantly associated with an increased chronic HCV infection risk among people ≥50 years (adjusted OR = 1.562, 95% CI: 1.079–2.262, *p* = 0.018), females (adjusted OR = 1.667, 95% CI: 1.145–2.429, *p* = 0.008), ALT <40 U/L (adjusted OR = 1.532, 95% CI: 1.074–2.286, *p* = 0.018), and AST <40 U/L (adjusted OR = 1.552, 95% CI: 1.095–2.201, *p* = 0.014). Additionally, considering data heterogeneity in different subgroups, we performed a heterogeneity test. Results showed no significant heterogeneity between groups (*p* > 0.05).

**Table 3:** Stratified analysis the association of rs9525641 with HCV chronicity.

**Subgroups**	**Group A**	**Group B**	**Group C**	**OR (95%CI)^**a**^**	***P* ^**a**^**	***P^***b***^***
	***n* (CC/CA/AA)**	***n* (CC/CA/AA)**	***n* (CC/CA/AA)**			
Age						
<50	145/284/126	51/100/28	80/155/65	1.482(0.900–2.442)	0.122	0.872
≥50	261/484/220	93/160/54	129/210/116	**1.562 (1.079**–**2.262)**	**0.018**	
Gender						
Male	179/300/133	58/92/33	81/126/64	1.330 (0.823–2.150)	0.245	0.474
Female	277/468/213	86/168/49	128/239/117	**1.667 (1.145**–**2.429)**	**0.008**	
ALT (U/L)						
<40	383/722/326	115/203/63	124/211/100	**1.532 (1.074**–**2.186)**	**0.018**	0.884
≥40	21/37/16	29/56/19	85/152/81	1.455 (0.826–2.562)	0.176	
AST (U/L)						
<40	380/729/321	112/213/65	130/210/106	**1.552 (1.095**–**2.201)**	**0.014**	0.736
≥40	22/30/21	30/43/17	79/147/73	1.373 (0.751–2.510)	0.303	
High-risk population						
HD	135/286/131	26/44/17	21/37/17	1.337 (0.610–2.928)	0.468	0.614
IVDU	46/93/38	37/74/15	41/67/32	2.263 (1.152–4.445)	0.018	
PBD	225/389/177	81/142/50	147/261/132	1.419 (0.983–2.047)	0.062	
HCV genotypes						0.708
1b	—	7/24/9	72/99/51	0.996 (0.347–2.858)	0.994	
Non-1b	—	38/54/19	57/136/58	1.302 (0.643–2.637)	0.464	

*CI, confidence interval; HCV, hepatitis C virus; OR, odds ratio; HD, hemodialysis patients; IVDU, Intravenous drug user; PBD, paid blood donors; Non-1b, viral strains other than 1b, including genotype 1a, 2, and 3 (either solely or mixed infection).*

*Group A: controls; Group B: spontaneous clearance subjects; Group C: persistent infection patients. Group (B+C): Infected individuals.*

*^a^The P-value, OR and 95% CIs of Group C vs. Group B were calculated on the basis of the logistic regression model, adjusted by gender, age, high-risk population.*

*^b^P-value for the heterogeneity test.*

*Bold type indicates statistically significant results*.

### Bioinformatics Analysis

The rs9525641 genotype had a Regulome DB score of 5, suggesting its potential functions such as transcription factor binding or DNase peak. Based on the HaploReg database, rs9525641 overlaps promoter histone marks, enhancer histone marks, and DNase and FXR motifs. Furthermore, 22 SNPs were in linkage disequilibrium with rs9525641 in the Asian population (*r*^2^ > 0.8) (available at HaploReg database). Results of the 22 SNPs are presented in Supplementary Table 3. The in?uence of those SNPs on the RANKL mRNA secondary structure was predicted using the RNAfold Web Server. Six SNPs presented local structure changes (rs17458177, rs1325799, rs17536328, rs7984870, rs9533155, and rs3742257) (Supplementary Figures 1–6). Moreover, rs17458177-C and -T alleles showed a difference in the lowest free energy (−18.40 vs. −18.90 kcal/mol), suggesting that mutations might affect RANKL transcription. Specific information for the other SNPs can be found in the Supplementary Figures 1–6.

## Discussion

The RANKL message is detected in the peripheral lymph nodes and bone marrow, thymus, spleen, Peyer's patches, brain, heart, skin, skeletal muscle, kidney, liver, lung, and mammary tissues (12). The RANKL/RANK system has been shown to play a critical role in the immune system, including lymph-node development, lymphocyte differentiation, dendritic cell survival, and T-cell activation and tolerance induction (13). RANKL can regulate lymph-node organogenesis, T- and B-lymphocyte development, and osteoclast differentiation. Some studies also indicated that RANKL regulates the thymus microenvironment by autoimmune regulators expression activation (14). Additionally, at the molecular level, RANK interacts with RANKL to activate the transcription factor NF-κB along with TNF receptor-related factor family signaling molecules (15). Considering that the NF-κB function is related to RANKL/RANK, and NF-κB has been linked to chronic hepatitis C (16–18), we hyphothesized that the RANKL/RANK pathway polymorphisms would affect the HCV infection outcome.

RANKL, RANK, and OPG are encoded by *TNFSF11* (gene map locus 13q14), *TNFRSF11A* (gene map locus 18q22.1), and *TNFRSF11B* (gene map locus 8q24), respectively (19). The *TNFSF11* gene structure is highly conserved among mammals, consisting of five exons that span 33.9 kb in humans (10). SNPs located near *TNFSF11, TNFRSF11A*, and *TNFRSF11B* have been reported to be closely associated with Paget's disease (20), osteoporotic fractures (21), cardiovascular diseases (22), ankylosing spondylitis (11), and breast (23), and esophageal cancers (24). In this study, we showed that subjects who carried the rs9525641-C allele were more likely to develop HCV chronicity than those with the rs9525641-T allele. Furthermore, in the stratification analyses based on age, gender, and high-risk population, we found that the rs9525641-C allele was associated with HCV chronicity among elders, females, and persons with ALT and AST <40 U/L. Interestingly, RANKL can cause various degenerative bone diseases, such as rheumatoid arthritis and osteoporosis. These diseases are mostly female. Our research shows that RANKL is related to the chronicity of HCV in women. This suggests to some extent the combined effect of gender factors and the RANKL system in disease progression, which is worthy of further discussion. However, the heterogeneity test showed no significant heterogeneity in any pair-wise comparison (*p* > 0.05), indicating that these variables did not materially affect the results.

Although rs9525641 is located in the *TNFSF11* intronic region, this variant might play an important role in gene transcription regulation or might be in linkage disequilibrium with other functional SNPs, such as rs17458177, rs1325799, rs17536328, rs7984870, rs9533155, and rs3742257. We also calculated the degree of linkage between the candidate SNPs, *r*^2^ < 0.1 indicates that there is no linkage disequilibrium among these three SNPs. The bioinformatics analysis indicated that these variants could regulate gene transcription, mRNA export, and protein translational efficiency. In the present study, we found two possible *TNFSF11* biological processes using the STRING database website prediction (https://string-db.org/cgi/network.pl?taskId=kGEafB5GcPa7:) extracellular signal-regulated kinase 1 (ERK1) and ERK2 cascade positive regulation via *TNFSF11*-mediated signaling; and the tumor necrosis factor-mediated signaling pathway. It has been reported that the HCV non-enveloped particles' intriguing cellular internalization properties can activate the ERK1/2 pathway that could be important in the HCV life cycle and infection pathogenesis (25). Additionally, Fletcher et al. reported that several TNF superfamily members – including TNF-α, TNF-β, TWEAK, and LIGHT – can promote HCV entry via NF-κB-mediated activation of myosin light chain kinase and tight junctions disruption (26). Therefore, *TNFSF11* may be involved in these pathways and contribute to the hepatitis C chronic process. However, these hypotheses are based on computer simulations and functional evaluations using biological assays. Thus, it should be warranted in future studies.

RANK is an intrinsic hematopoietic cell surface receptor that stimulates NF-κB receptor activation, plays a central role in T and dendritic cells, and promotes lymph node development (27)^.^ Previously, several studies have found that the TNFRSF11 rs8680340 was closely related to the anti-citrullinated peptide antibody and age at natural menopause (28). Additionally, the *TNFRSF11B* rs2073618 was significantly associated with Type 2 Diabetes (29), rheumatoid arthritis (30), and volumetric bone mineral density (31). However, in this study, no significant association was observed between *TNFRSF11A* rs8086340, *TNFRSF11B* rs2073618, and HCV infection outcome.

Our study also has some limitations. First, this study was performed with the Chinese Han population, requiring further reproduction in different ethnic populations. Second, we did not collect immune markers data in the RANKL/RANK pathway and could not check the association between immune markers data and the target SNPs. Third, we did not collect complete virus subtypes and viral load data and could not check the association between virus data and the target SNPs. Hence, we should improve the collection of this part of the data in the later stagethe and possible immune mechanism requires to be further studied and verified. Furthermore, we did not explore how the functional mechanisms of these SNPs affect HCV chronicity using molecular biology approaches. Then, functional studies are required in the future to explore how these polymorphisms impact chronic HCV infection.

Overall, our findings suggested that the rs9525641 *TNFSF11* polymorphism might affect HCV chronicity in a high-risk Chinese population. Larger well-designed epidemiological studies with ethnically diverse populations and functional evaluations are warranted to confirm these findings before the effect of these variants can be fully and accurately evaluated.

## Data Availability Statement

The original contributions presented in the study are included in the article/Supplementary Material, further inquiries can be directed to the corresponding author/s.

## Ethics Statement

The studies involving human participants were reviewed and approved by the Ethics Committee of Nanjing Medical University. The patients/participants provided their written informed consent to participate in this study.

## Author Contributions

SY and R-BY: conceptualization. Y-QH: formal analysis, investigation, and writing–original draft. PH: funding acquisition and methodology. Y-DW and J-JW: resources. J-JW and FZ: software. SY: supervision. Y-QH and X-YY: writing–review and editing. All authors contributed to the article and approved the submitted version.

## Funding

This study was supported in part by National Natural Science Foundation of China (no. 81703273), Natural Science Foundation of Jiangsu Province (no. BK20171054), Natural Science Foundation of Yunnan Province (no. 2019FA005), and the Priority Academic Program Development of Jiangsu Higher Education Institutions (PAPD).

## Conflict of Interest

The authors declare that the research was conducted in the absence of any commercial or financial relationships that could be construed as a potential conflict of interest.

## Publisher's Note

All claims expressed in this article are solely those of the authors and do not necessarily represent those of their affiliated organizations, or those of the publisher, the editors and the reviewers. Any product that may be evaluated in this article, or claim that may be made by its manufacturer, is not guaranteed or endorsed by the publisher.

## Supplementary Material

Supplementary Figure 1 The influence of rs17458177 on the RANKL mRNA secondary structures. The arrow indicates the position of the mutation (50 bases upstream and 50 bases downstream of the mutation). The minimum free energies for the C and T allele of rs17458177 were estimated at −18.40 and −18.90 kcal/mol, respectively, by RNAfold Wed Server.

Supplementary Figure 2 The influence of rs1325799 on the RANKL mRNA secondary structures. The arrow indicates the position of the mutation (50 bases upstream and 50 basesdownstream of the mutation). The minimum free energies for the G and A allele of rs1325799 were estimated at −11.80 and −14.00 kcal/mol, respectively, by RNAfold Wed Server.

Supplementary Figure 3 The influence of rs17536328 on the RANKL mRNA secondary structures. The arrow indicates the position of the mutation (50 bases upstream and 50 basesdownstream of the mutation). The minimum free energies for the C and T allele of rs17536328 were estimated at −17.4 and −16.60 kcal/mol, respectively, by RNAfold Wed Server.

Supplementary Figure 4 The influence of rs7984870 on the RANKL mRNA secondary structures. The arrow indicates the position of the mutation (50 bases upstream and 50 basesdownstream of the mutation). The minimum free energies for the G and C allele of rs7984870 were estimated at −14.40 and −15.50 kcal/mol, respectively, by RNAfold Wed Server.

Supplementary Figure 5 The influence of rs9533155 on the RANKL mRNA secondary structures. The arrow indicates the position of the mutation (50 bases upstream and 50 basesdownstream of the mutation). The minimum free energies for the C and G allele of rs9533155 were estimated at −14.80 and −15.50 kcal/mol, respectively, by RNAfold Wed Server.

Supplementary Figure 6 The influence ofrs3742257 on the RANKL mRNA secondary structures. The arrow indicates the position of the mutation (50 bases upstream and 50 basesdownstream of the mutation). The minimum free energies for the T and C allele of rs3742257 were estimated at −31.70 and −27.80 kcal/mol, respectively, by RNAfold Wed Server.

Supplementary Table 1 Probes and primers of investigated *TNFSF* and *TNFRSF* SNPs for Taman assay.

Supplementary Table 2 The detailed information on different RegulomeDB scores.

Supplementary Table 3 Annotation of variannts with stong linkage disequilibrim with SNP rs 9525641 in HaploRegV4.1.

## References

[1] WHO. Global Hepatitis Report, 2017. Geneva: WHO (2017).

[2] Rossi C Butt ZA Wong S Buxton JA Islam N Yu A. Hepatitis C virus reinfection after successful treatment with direct-acting antiviral therapy in a large population-based cohort. J Hepatol. (2018) 69:1007–14. 10.1016/j.jhep.2018.07.025 30142429

[3] Feng W Guo J Li M. RANKL-independent modulation of osteoclastogenesis. J Oral Biosci. (2019) 61:16–21. 10.1016/j.job.2019.01.001 30929797

[4] Zavala-Cerna MG Moran-Moguel MC Cornejo-Toledo JA Gonzalez-Montoya NG Sanchez-Corona J Salazar-Paramo M. Osteoprotegerin polymorphisms in a Mexican population with rheumatoid arthritis and generalized osteoporosis: a preliminary report. J Immunol Res. (2015) 2015:376197. 10.1155/2015/376197 26065000 PMC4433710

[5] Dostert C Grusdat M Letellier E Brenner D. The TNF family of ligands and receptors: communication modules in the immune system and beyond. Physiol Rev. (2019) 99:115–60. 10.1152/physrev.00045.2017 30354964

[6] Cooper S Erickson AL Adams EJ Kansopon J Weiner AJ Chien DY. Analysis of a successful immune response against hepatitis C virus. Immunity. (1999) 10:439–49. 10.1016/S1074-7613(00)80044-8 10229187

[7] Promrat K Liang TJ. Chemokine systems and hepatitis C virus infection: is truth in the genes of the beholders? Hepatology. (2003) 38:1359–62. 10.1053/jhep.2003.10008 14647045

[8] Gao B Hong F Radaeva S. Host factors and failure of interferon-alpha treatment in hepatitis C virus. Hepatology. (2004) 39:880–90. 10.1002/hep.20139 15057887

[9] Rehermann B Nascimbeni M. Immunology of hepatitis B virus and hepatitis C virus infection. Nat Rev Immunol. (2005) 5:215–29. 10.1038/nri1573 15738952

[10] Omar HS Shaker OG Nassar YH Marzouk SA ElMarzouky MS. The association between RANKL and osteoprotegerin gene polymorphisms with breast cancer. Mol Cell Biochem. (2015) 403:219–29. 10.1007/s11010-015-2352-z 25724681

[11] Wang CM Tsai SC Lin JC Wu YJ Wu J Chen JY. Association of genetic variants of RANK, RANKL, and OPG with ankylosing spondylitis clinical features in Taiwanese. Mediators Inflamm. (2019) 2019:8029863. 10.1155/2019/8029863 31015798 PMC6446096

[12] Anandarajah AP. Role of RANKL in bone diseases. Trends Endocrinol Metab. (2009) 20:88–94. 10.1016/j.tem.2008.10.007 19185505

[13] Xing L Schwarz EM Boyce BF. Osteoclast precursors, RANKL/RANK, and immunology. Immunol Rev. (2005) 208:19–29. 10.1111/j.0105-2896.2005.00336.x 16313338

[14] Sugiyama M Nakato G Jinnohara T Akiba H Okumura K Ohno H. Expression pattern changes and function of RANKL during mouse lymph node microarchitecture development. Int Immunol. (2012) 24:369–78. 10.1093/intimm/dxs002 22354913

[15] Riemann M Andreas N Fedoseeva M Meier E Weih D Freytag H. Central immune tolerance depends on crosstalk between the classical and alternative NF-?B pathways in medullary thymic epithelial cells. J Autoimmun. (2017) 81:56–67. 10.1016/j.jaut.2017.03.007 28385374

[16] Fakhir FZ Lkhider M Badre W Alaoui R Pineau P Ezzikouri S. The−94Ins/DelATTG polymorphism in NFkappaB1 promoter modulates chronic hepatitis C and liver disease progression. Infect Genet Evol. (2016) 39:141–6. 10.1016/j.meegid.2016.01.023 26827631

[17] Fan HZ Huang P Shao JG Tian T Li J Zang F. Genetic variation on the NFKB1 genes associates with the outcomes of HCV infection among Chinese Han population. Infect Genet Evol. (2018) 65:210–5. 10.1016/j.meegid.2018.07.031 30056167

[18] Tian T Wang J Huang P Li J Yu R Fan H. Genetic variations in NF-KappaB were associated with the susceptibility to hepatitis C virus infection among Chinese high-risk population. Sci Rep. (2018) 8:104. 10.1038/s41598-017-18463-y 29311624 PMC5758514

[19] Tu P Duan P Zhang RS Xu DB Wang Y Wu HP. Polymorphisms in genes in the RANKL/RANK/OPG pathway are associated with bone mineral density at different skeletal sites in post-menopausal women. Osteopor Int. (2015) 26:179–85. 10.1007/s00198-014-2854-7 25138264

[20] Ralston SH Taylor JP. Rare inherited forms of paget's disease and related syndromes. Calcif Tissue Int. (2019) 104:501–16. 10.1007/s00223-019-00520-5 30756140 PMC6779132

[21] Ye W Wang Y Hou S Mei B Liu X Huang H. USF3 modulates osteoporosis risk by targeting WNT16, RANKL, RUNX2, and two GWAS Lead SNPs Rs2908007 and Rs4531631. Hum Mutat. (2021) 42:37–49. 10.1002/humu.24126 33058301

[22] Singh MM Kumar R Tewari S Agarwal S. Investigation of OPG/RANK/RANKL genes as a genetic marker for cardiac abnormalities in Thalassemia major patients. Ann Hum Genet. (2017) 81:117–24. 10.1111/ahg.12189 28244588

[23] Ney JT Juhasz-Boess I Gruenhage F Graeber S Bohle RM Pfreundschuh M. Genetic polymorphism of the OPG gene associated with breast cancer. BMC Cancer. (2013) 13:40. 10.1186/1471-2407-13-40 23369128 PMC3563620

[24] Yin J Wang L Tang W Wang X Lv L Shao A. RANK Rs1805034 T>C polymorphism is associated with susceptibility of esophageal cancer in a Chinese population. PLoS ONE. (2014) 9:E101705. 10.1371/journal.pone.0101705 25019155 PMC4096509

[25] Katsarou K Lavdas AA Tsitoura P Serti E Markoulatos P Mavromara P. Endocytosis of hepatitis C virus non-enveloped capsid-like particles induces MAPK-ERK1/2 signaling events. Cell Mol Life Sci. (2010) 67:2491–506. 10.1007/s00018-010-0351-5 20358251 PMC11115770

[26] Fletcher NF Clark AR Balfe P McKeating JA. TNF superfamily members promote hepatitis C virus entry via an NF-KappaB and myosin light chain kinase dependent pathway. J Gen Virol. (2017) 98:405–12. 10.1099/jgv.0.000689 27983476 PMC5797950

[27] Ono T Hayashi M Sasaki F Nakashima T. RANKL biology: bone metabolism, the immune system, and beyond. Inflamm Regen. (2020) 40:2. 10.1186/s41232-019-0111-3 32047573 PMC7006158

[28] Lu Y Liu P Recker RR Deng HW Dvornyk V. TNFRSF11A and TNFSF11 are associated with age at menarche and natural menopause in white women. Menopause. (2010) 17:1048–54. 10.1097/gme.0b013e3181d5d523 20531232 PMC2939156

[29] Pleskovič A Ramuš SM PraŽnikar ZJ Šantl Letonja M Cokan Vujkovac A Gazdikova K. Polymorphism Rs2073618 of the osteoprotegerin gene as a potential marker of subclinical carotid atherosclerosis in caucasians with type 2 diabetes mellitus. Vasa. (2017) 46:355–62. 10.1024/0301-1526/a000640 28593808

[30] Chen Y Yang Y Liu G. Association between osteoprotegerin gene polymorphisms and rheumatoid arthritis susceptibility: a meta-analysis. Arch Med Res. (2016) 47:134–41. 10.1016/j.arcmed.2016.05.001 27156396

[31] Bonfá AC Seguro LP Caparbo V Bonfá E Pereira RM. RANKL and OPG gene polymorphisms: associations with vertebral fractures and bone mineral density in premenopausal systemic lupus erythematosus. Osteoporos Int. (2015) 26:1563–71. 10.1007/s00198-015-3029-x 25609157

