# Supplementary figures and images for: Genetic Mutations in TNFSF11 Were Associated With the Chronicity of Hepatitis C Among Chinese Han Population
Source: Front Med (Lausanne). 2021 Oct 1;8:743406. doi: 10.3389/fmed.2021.743406 (PMC8517249; doi:10.3389/fmed.2021.743406)

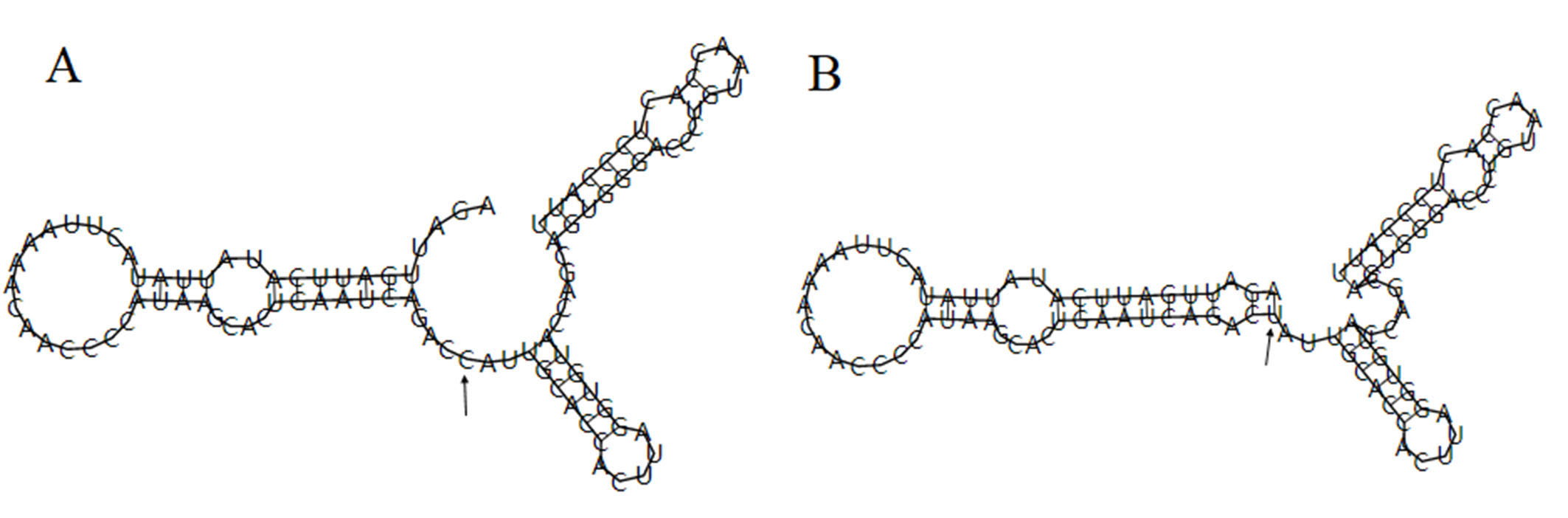

Supplement: Supplementary Figure 1 — The influence of rs17458177 on the RANKL mRNA secondary structures. The arrow indicates the position of the mutation (50 bases upstream and 50 bases downstream of the mutation). The minimum free energies for the C and T allele of rs17458177 were estimated at −18.40 and −18.90 kcal/mol, respectively, by RNAfold Wed Server. [file Image_1.JPEG]

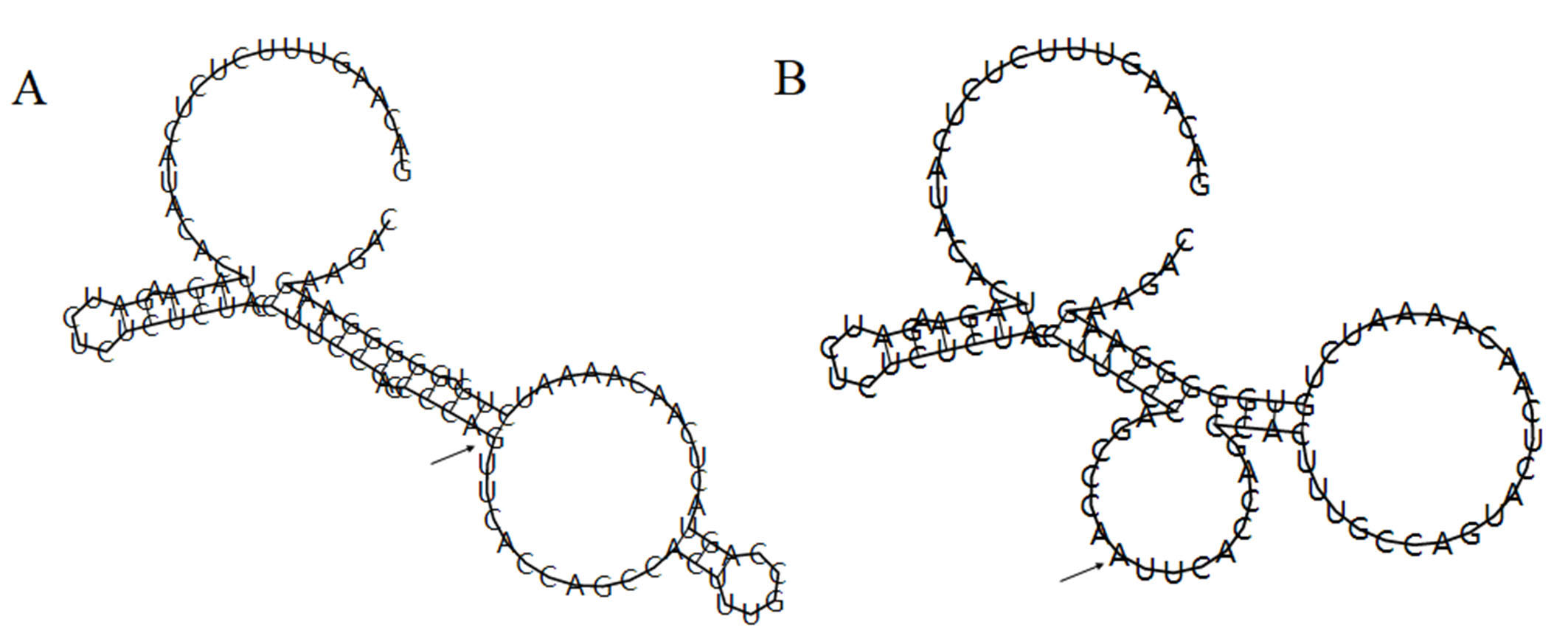

Supplement: Supplementary Figure 2 — The influence of rs1325799 on the RANKL mRNA secondary structures. The arrow indicates the position of the mutation (50 bases upstream and 50 basesdownstream of the mutation). The minimum free energies for the G and A allele of rs1325799 were estimated at −11.80 and −14.00 kcal/mol, respectively, by RNAfold Wed Server. [file Image_2.JPEG]

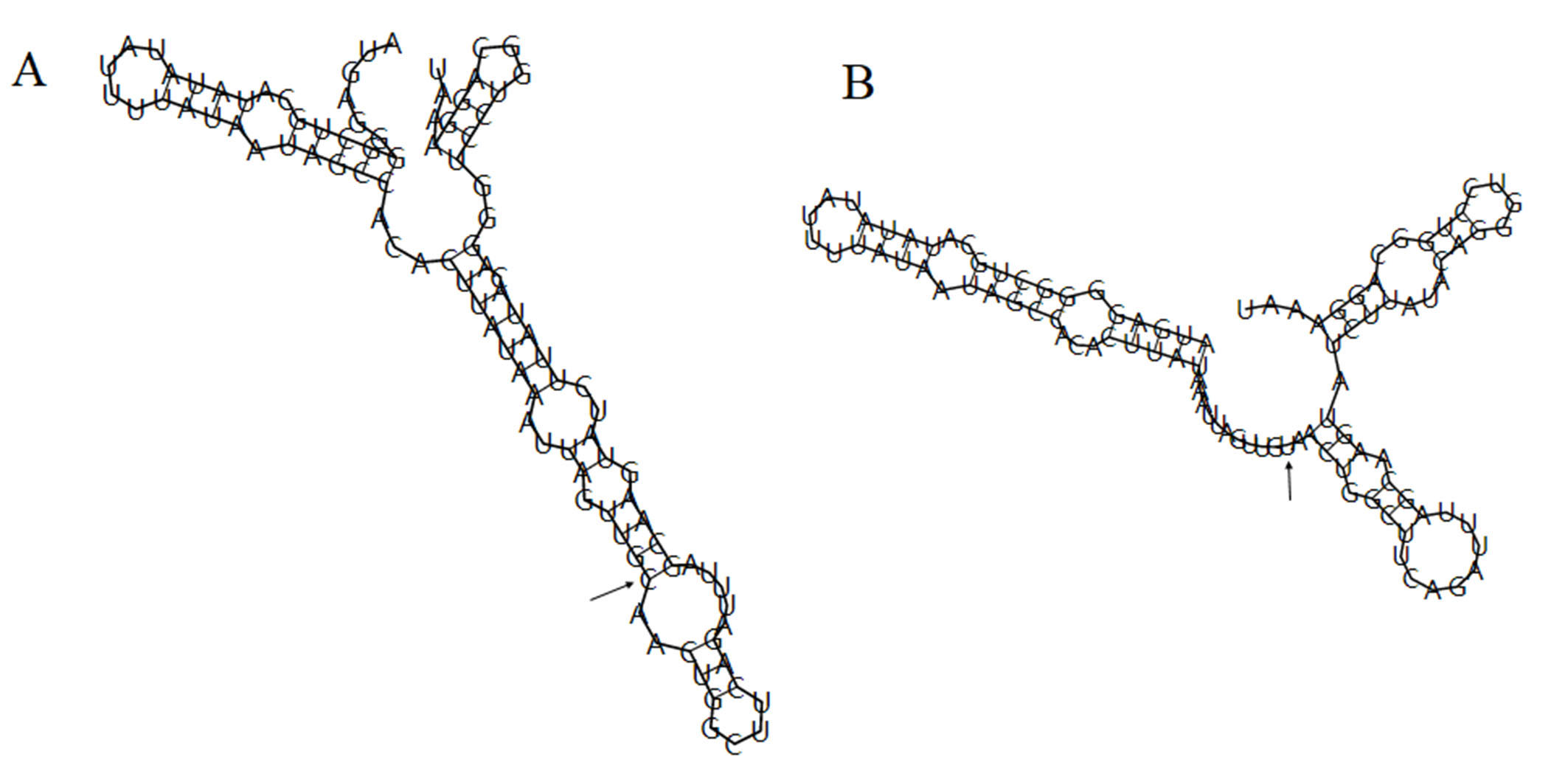

Supplement: Supplementary Figure 3 — The influence of rs17536328 on the RANKL mRNA secondary structures. The arrow indicates the position of the mutation (50 bases upstream and 50 basesdownstream of the mutation). The minimum free energies for the C and T allele of rs17536328 were estimated at −17.4 and −16.60 kcal/mol, respectively, by RNAfold Wed Server. [file Image_3.JPEG]

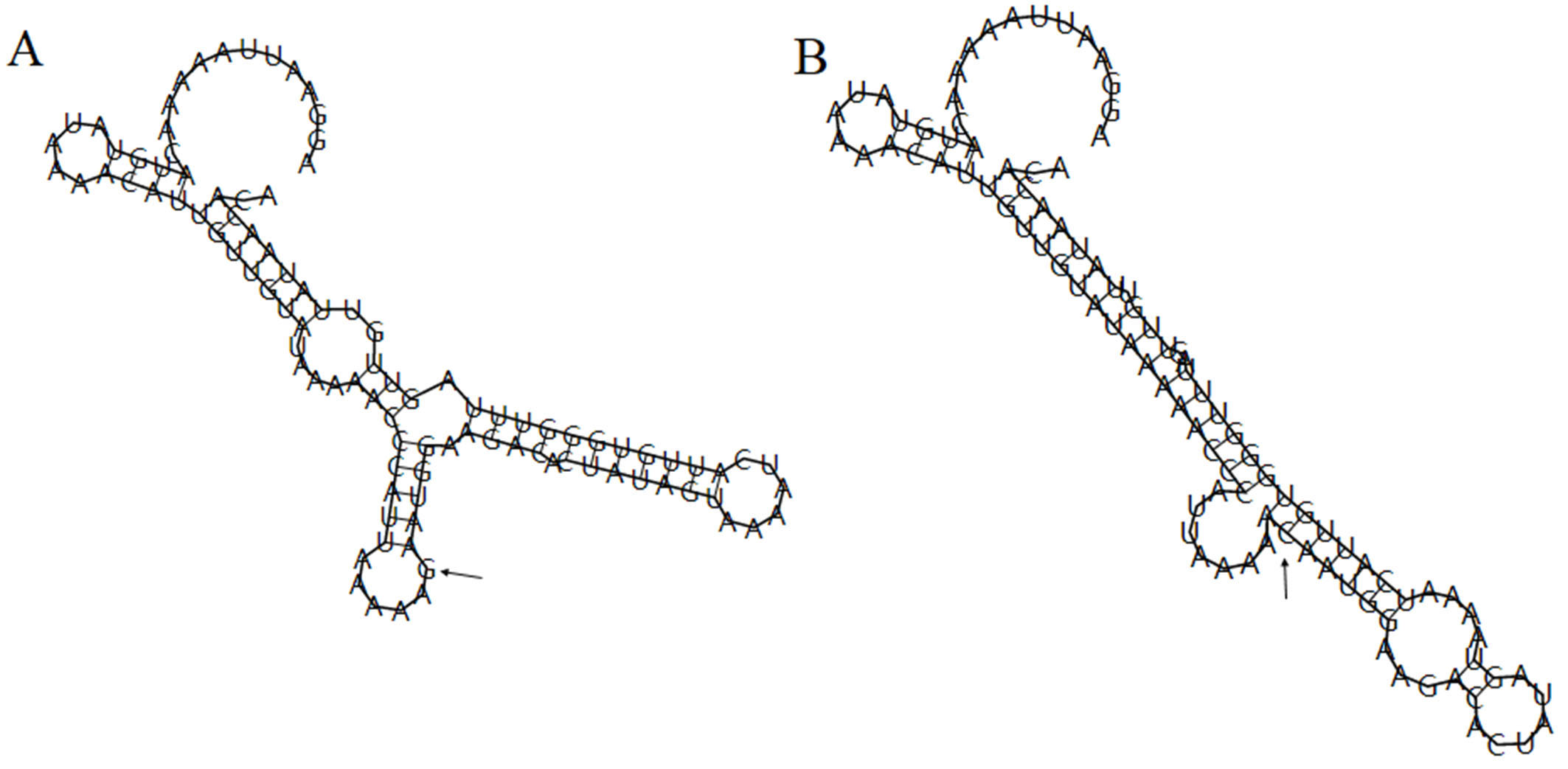

Supplement: Supplementary Figure 4 — The influence of rs7984870 on the RANKL mRNA secondary structures. The arrow indicates the position of the mutation (50 bases upstream and 50 basesdownstream of the mutation). The minimum free energies for the G and C allele of rs7984870 were estimated at −14.40 and −15.50 kcal/mol, respectively, by RNAfold Wed Server. [file Image_4.JPEG]

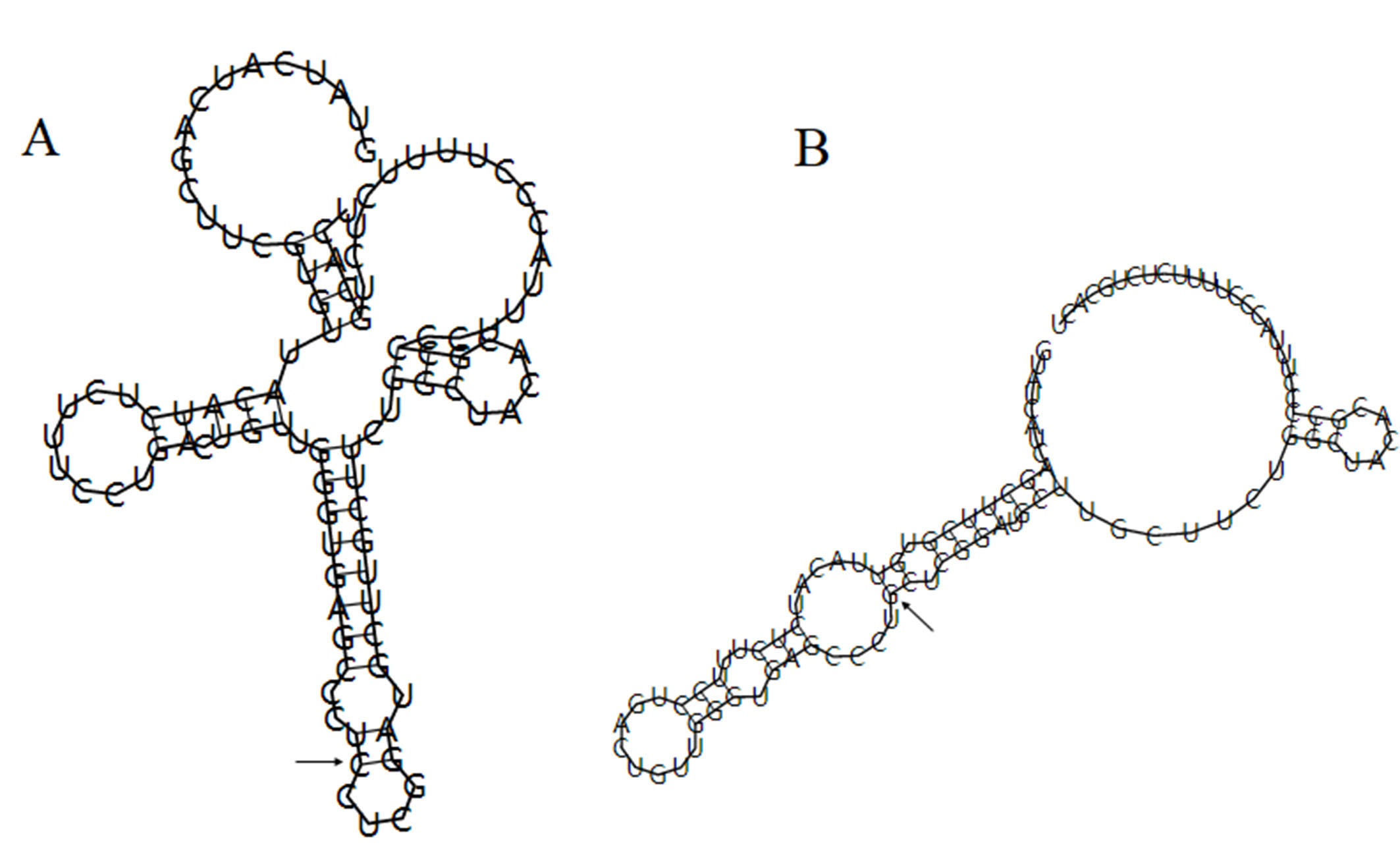

Supplement: Supplementary Figure 5 — The influence of rs9533155 on the RANKL mRNA secondary structures. The arrow indicates the position of the mutation (50 bases upstream and 50 basesdownstream of the mutation). The minimum free energies for the C and G allele of rs9533155 were estimated at −14.80 and −15.50 kcal/mol, respectively, by RNAfold Wed Server. [file Image_5.JPEG]

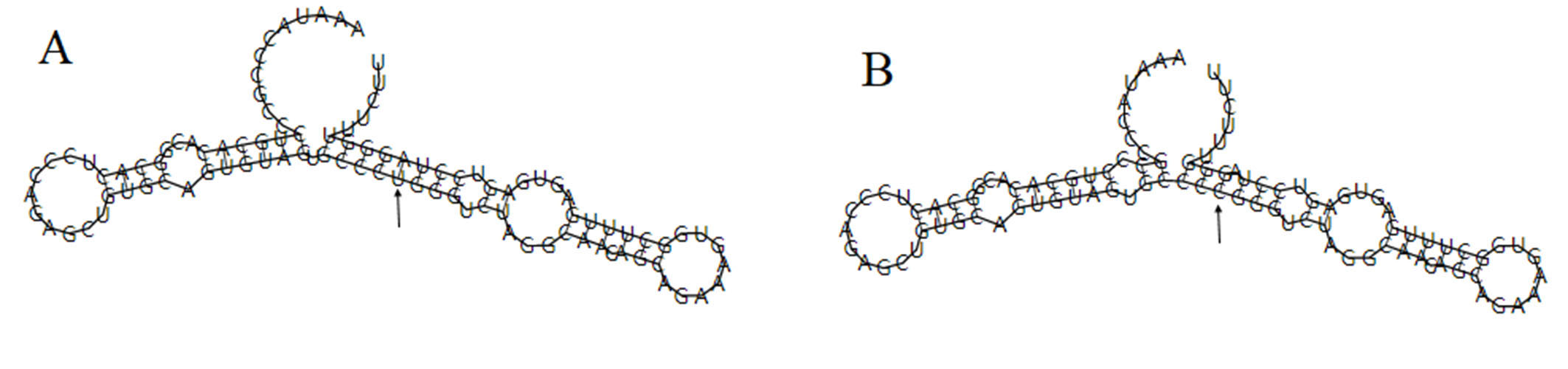

Supplement: Supplementary Figure 6 — The influence ofrs3742257 on the RANKL mRNA secondary structures. The arrow indicates the position of the mutation (50 bases upstream and 50 basesdownstream of the mutation). The minimum free energies for the T and C allele of rs3742257 were estimated at −31.70 and −27.80 kcal/mol, respectively, by RNAfold Wed Server. [file Image_6.JPEG]
