# Supplementary material for: Genetic Mutations in TNFSF11 Were Associated With the Chronicity of Hepatitis C Among Chinese Han Population
Source: Front Med (Lausanne). 2021 Oct 1;8:743406. doi: 10.3389/fmed.2021.743406 (PMC8517249; doi:10.3389/fmed.2021.743406)
Supplement: Supplementary Table 1 — Probes and primers of investigated TNFSF and TNFRSF SNPs for Taman assay. [file Table_1.DOCX]

**Table S1** Probes and primers of investigated *TNFSF* and *TNFRSF* SNPs for TaqMan assay

| SNPs (Allele) | Gene | Region | MAF^a/b^ | *P^c^* | *P^d^* | TaqMan-MGB probe/primers sequences (5’-3’) |
| --- | --- | --- | --- | --- | --- | --- |
| rs9525641 | TNFSF11 | intron 1 | 0.480/0.439 | 0.637 | 0.053 | Probe-A: HEX-TGGGTTAAAGAAGACG-MGB |
| (T>C) |  |  |  |  |  | Probe-G: FAM-ATGGGTTAAGGAAGACG-MGB |
|  |  |  |  |  |  | Forward primer: AGCTGTCTTTCTGGAGGTCCAA |
|  |  |  |  |  |  | Reverse primer: GGTGCAATCCTTATCCTTTAGCA |
| rs8086340 | TNFRSF11A | intron 1 | 0.351/0.329 | 0.250 | 0.274 | Probe-G: FAM-TTTCTCCAGAAGATAGAG-MGB |
| (G>C) |  |  |  |  |  | Probe-C: HEX-CTCCAGAAGATACAGTTT-MGB |
|  |  |  |  |  |  | Forward primer: TCACTCGCCTTTATCTCGAAAAA |
|  |  |  |  |  |  | Reverse primer: ACAGCAGAACTTCGTAAAGAGATTGA |
| rs2073618 | TNFRSF11B | Missense | 0.255/0.239 | 0.113 | 0.056 | Probe-G: HEX-CCACAATGAACAAGTT-MGB |
| (G>C) |  |  |  |  |  | Probe-C: FAM-CCACAATGAACAACTT-MGB |
|  |  |  |  |  |  | Forward primer: TCCAAGCCCCTGAGGTTTC |
|  |  |  |  |  |  | Reverse primer: AGGGACTTACCACGAGCGC |

Abbreviations: SNPs, single nucleotide polymorphisms; MAF, minor allele frequency; TNFSF, tumor necrosis factor superfamily; TNFRSF, tumor necrosis factor receptor superfamily.

^a^ minor allele frequencies in control group (Group A).

^b^ minor allele frequencies from HapMap of Han Chinese in Beijing, China (CHB) or East Asia (EAS). (available at

<https://www.ncbi.nlm.nih.gov/snp> ).

^c^ *P* value of Hardy-Weinberg equilibrium for SNPs among control group (Group A).

^d^ *P* value of Hardy-Weinberg equilibrium for SNPs among spontaneous HCV clearance subjects (Group B).

**Table S2** The detailed information on different RegulomeDB scores

| Score | Supporting data |
| --- | --- |
| 1a | eQTL + TF binding + matched TF motif + matched DNase Footprint + DNase peak |
| 1b | eQTL + TF binding + any motif + DNase Footprint + DNase peak |
| 1c | eQTL + TF binding + matched TF motif + DNase peak |
| 1d | eQTL + TF binding + any motif + DNase peak |
| 1e | eQTL + TF binding + matched TF motif |
| 1f | eQTL + TF binding / DNase peak |
| 2a | TF binding + matched TF motif + matched DNase Footprint + DNase peak |
| 2b | TF binding + any motif + DNase Footprint + DNase peak |
| 2c | TF binding + matched TF motif + DNase peak |
| 3a | TF binding + any motif + DNase peak |
| 3b | TF binding + matched TF motif |
| 4 | TF binding + DNase peak |
| 5 | TF binding or DNase peak |
| 6 | other |

eQTL: expression Quantitative Trait Loci; TF: transcription factor; DNase: deoxyribonuclease.

**Table S3** Annotation of variannts with stong linkage disequilibrim with SNP rs 9525641 in HaploRegV4.1

| Chr | Pos  (hg19) | LD  (𝑟^2^) | Variant | Ref | Alt | ASN freq | Promoter histone marks | DNAse | Proteins bound | Motifs changed |
| --- | --- | --- | --- | --- | --- | --- | --- | --- | --- | --- |
| 13 | 42543635 | 0.90 | rs1853856 | T | G | 0.42 |  | 6 tissues |  | ATF3,GR,YY1 |
| 13 | 42543764 | 0.90 | rs9533146 | G | A | 0.42 |  |  |  | 6 altered motifs |
| 13 | 42554441 | 0.83 | rs9525638 | T | C | 0.39 |  | VAS |  | 7 altered motifs |
| 13 | 42556230 | 0.96 | rs9566987 | A | G | 0.42 |  |  |  | Egr-1 |
| 13 | 42559230 | 0.99 | rs17458177 | C | T | 0.42 |  |  |  | 4 altered motifs |
| 13 | 42559279 | 0.99 | rs66939206 | T | C | 0.42 |  | IPSC, BLD |  | AP-2rep,CACD,Klf7 |
| 13 | 42559396 | 0.99 | rs942915 | C | T | 0.42 |  |  |  | 5 altered motifs |
| 13 | 42559396 | 0.85 | rs17536280 | C | T | 0.38 |  | SKIN,SKIN,BLD | CFOS | GR |
| 13 | 42564913 | 0.85 | rs1325798 | C | T | 0.38 |  |  |  |  |
| 13 | 42564929 | 0.85 | rs1325799 | G | A | 0.38 |  |  |  |  |
| 13 | 42565284 | 1 | rs7997264 | T | C | 0.42 |  |  |  | 4 altered motifs |
| 13 | 42565966 | 0.85 | rs9533154 | C | T | 0.38 |  |  |  | 4 altered motifs |
| 13 | 42568893 | 0.85 | rs17536328 | C | T | 0.38 |  |  |  | 7altered motifs |
| 13 | 42569392 | 1 | rs7981403 | C | T | 0.42 |  |  |  | 4 altCACD,p300 ered motifs |
| 13 | 42571183 | 0.85 | rs7325635 | G | A | 0.38 |  |  |  | CACD,p300 |
| 13 | 42572346 | 1 | rs7984870 | G | C | 0.42 |  |  |  | 9 altered motifs |
| 13 | 42573485 | 0.85 | rs9533155 | C | G | 0.38 | BLD | LNG |  | Pax-5 |
| 13 | 42573535 | 1 | rs9533156 | T | C | 0.42 | BLD | ESC |  | Foxo,HNF4 |
| 13 | 42574429 | 1 | rs2296533 | T | C | 0.42 | 15 tissues | IPSC, THYM |  | 26 altered motifs |
| 13 | 42585668 | 0.93 | rs9525644 | G | A | 0.43 |  | LNG |  | 9 altered motifs |
| 13 | 42591246 | 0.91 | rs7330889 | G | A | 0.43 |  | LNG, CRVX |  | Dbx1,HDAC2,Irf |
| 13 | 42599062 | 0.90 | rs3742257 | T | C | 0.56 |  |  |  | LBP-1 |

Chr: chromosome, Pos: position, LD: linkage disequilibrium in Asian population, Ref: reference, Alt: alternative, freq: frequency, MUS: musculus, and

IPSC: induced pluripotent stem cell.
